# Trends in Open Aortic Valve Surgery in the United States: Descriptive Analysis of Sociodemographic Factors in Patient Selection

**DOI:** 10.1186/s12893-026-03621-9

**Published:** 2026-02-25

**Authors:** Élan Burton, Ben Marafino, Katharine Casselman Pines, Tim Morrison, Matthew Chuan-Tai Groeneveld, Michael Baiocchi

**Affiliations:** 1 Department of Cardiothoracic Surgery, Stanford University School of Medicine, 870 Quarry Rd Ext 2nd Floor Falk Bldg, CA Palo Alto, 94304 USA; 2 Division of Research, Kaiser Permanente, Oakland, CA USA; 3 Department of Health Systems Science, Kaiser Permanente Bernard J. Tyson School of Medicine, Pasadena, CA USA; 4 Department of Statistics, Stanford University, Palo Alto, CA USA; 5 Department of Epidemiology and Population Health, Stanford University School of Medicine, Palo Alto, CA USA

**Keywords:** aortic valve replacement, minimally invasive surgery, health equity, health disparity

## Abstract

**Background:**

Minimally invasive surgical approaches offers patient benefit, such as expedited recovery, and could reduce hospital cost. This study examines how sociodemographic factors influences surgical approach for aortic valve surgery.

**Methods:**

We used data from The Society of Thoracic Surgeons’ database to model selection into minimally invasive surgery vs. traditional sternotomy.

Pair-matches were created between two identity types: male-vs-female sex and White-vs-Black race. Patients were matched on facility and other covariates. These pair-matches were summarized using generalized linear mixed models (logit-link), regressing surgery type on the relevant identity, with random effects for facility and matched pair. Additionally, a regional analysis summarizing variation in the mortality-risk profiles of patients was conducted.

**Results:**

From 2015 to 2020, of the patients that met inclusion criteria, 68,956 patients underwent traditional sternotomy and 23,811 underwent minimally invasive surgery. For matched pairs of the examined covariate, the null hypothesis was that each patient would have the same odds of receiving each surgical approach.

Our models estimate the odds ratio for receiving the minimally invasive surgery are 1.13 and 1.56 times higher for female and White patients respectively (both p-values <= 0.005).

We also identified regional variation across levels of mortality-risk score and race.

**Conclusions:**

Our study demonstrates a pattern of variation in sorting in minimally invasive surgical aortic valve replacement vs traditional sternotomy via patient sex and race. These findings infer non-medical features guide patient candidacy for surgical approach, even within the same facility.

**Supplementary Information:**

The online version contains supplementary material available at 10.1186/s12893-026-03621-9.

## Introduction

Aortic stenosis (AS) has become the third most prevalent cardiovascular disease [1, 2]. Patients with AS who do not receive aortic valve replacement (AVR) have a 50% mortality risk at two years while those who receive an AVR have significantly better survival rates at 1 and 3 years [2–4]. African American patients have historically undergone fewer traditional full sternotomy surgical aortic valve replacements (SAVRs) for AS than White patients, which has been attributed to lower prevalence of AS in African Americans, thus decreased need for surgical intervention [4, 5]. African American and Latino patients who undergo SAVR typically do so under emergent, urgent, and/or non-elective admissions and at a younger age than their White counterparts [5–7]; with African American patients having greater crude mortality rates, longer hospitalization duration, increased hospitalization cost, and a greater number of discharges to skilled nursing facilities than White patients [5]. Though females have greater long-term survival vs. men, similarly, females have been reported to have increased postoperative complications and symptomology [8, 9].

Before the introduction of transcatheter approaches, the bulk of AVRs had been performed through a full sternotomy, though minimally invasive surgical aortic valve replacement (MISAVR) has been accessible in the United States since 1996, also as an alternative [10, 11]. Moreover, MISAVR procedures typically result in less post-operative pain, reduced ventilation times, decreased blood loss, and reduced need for blood transfusion [11–17] Studies have also shown obese patients have benefited from MISAVR, with lower rates of renal failure and mortality in a randomized control trial of MISAVR vs. full sternotomy [17, 18].

Merk et al. performed a propensity matched comparison of patients undergoing MISAVR vs. SAVR and reported good short and long-term survival results with both approaches [19]. Despite the similar survival rates and potential recovery benefits associated with MISAVR, a paucity of data exists on whether under-represented minority (URM) and/or female patients are receiving minimally invasive procedures compared to traditional open procedures at the same rate as their White, male counterparts. Coupled with the existence of health outcome disparities in AS treatment among female and African American patients, it is important to carefully describe the existence of variations in utilization of surgical approach as there may be unaccounted for burden to patients that already experience disparate care. Following a definition used by the World Health Organization for health inequity, we focus on how disparate access or health status between groups of people can lead to hardships socially and economically [20] For instance, LaPar et al. found that patients that were un-/under-insured had a higher rate of complications post cardiac valve surgery, which could lead to patients suffering further financial setbacks due to prolonged recovery [21].

Reflecting on the data presented above, our study aims to examine the influence of patient sociodemographic factors (race and sex) on patient selection in type of operative approach used to perform open aortic valve surgery (SAVR vs. MISAVR) to determine if selection bias exists. We attempt to isolate variation in selection between SAVR and MISAVR both (i) within the same surgical facility (within-site), and more generally (ii) within-region.

## Methods

Our study was approved by the Stanford University Institutional Review Board on June 4, 2021, eProtocol#52,045 IRB 61 registration 4947. We used data from The Society of Thoracic Surgeons’ database collected in the United States between 2015 and 2020 to model selection into minimally invasive surgery vs. traditional full sternotomy. We did not examine patients undergoing transcatheter aortic valve replacement (TAVR) as this data is held by a different database and is not accessible under the Participant User File Research Program (PUF) agreement. Additionally, this manuscript aimed to examine selection of patients for open surgical approaches.

### Human ethics and consent to participate declarations: not applicable

The Participant User File Research Program provides deidentified data for secondary data analysis purposes thus the Stanford IRB did not require obtaining consent.

### Data availability

The data in this manuscript was provided by a third party (The Society of Thoracic Surgeons’ National Database Participant User File Research Program) under legal contract and by permission. Data will be shared upon reasonable request to the corresponding author with permission of the third party listed.

### Treatment levels: definition of MISAVR and SAVR

Using the STS coding, we defined SAVR as an AVR with traditional full sternotomy and MISAVR as an AVR with the coding of: partial sternotomy, right or left parasternal incision, left or right thoracotomy, limited (right, left, or bilateral) thoracotomy, port access.

### Inclusion/exclusion

The primary inclusion criterion was a patient receiving either SAVR or MISAVR. Our population consisted of adult patients with isolated aortic valve disease with stenosis being the predominant disease process, undergoing aortic valve replacement under elective or urgent conditions as defined by the STS Data Specifications 2004 [22]. Patients with the following criteria were excluded from our analysis: under the age of 18, history of (h/o) any open heart surgery (re-do sternotomy/thoracotomy), h/o mediastinal radiation, presence of moderate or severe COPD, diagnosis of active endocarditis, diagnosis of primary aortic valve insufficiency, patients undergoing other concomitant heart surgery (e.g. multi-valve replacement, valve with coronary artery bypass grafting, root replacements, ascending aorta replacements, etc.), patients on inotropic, ventilatory support, who received thrombolytic treatment 48 h prior to surgery, or patients that were classified as emergency cases as defined by STS Data Specifications 2004 [22]. Per the inclusion/exclusion criteria and the PUF agreement, we only received data that satisfied inclusion criteria. Thus, 100% of the patients included in this analysis were either elective or urgent cases.

**Fig. 1:**
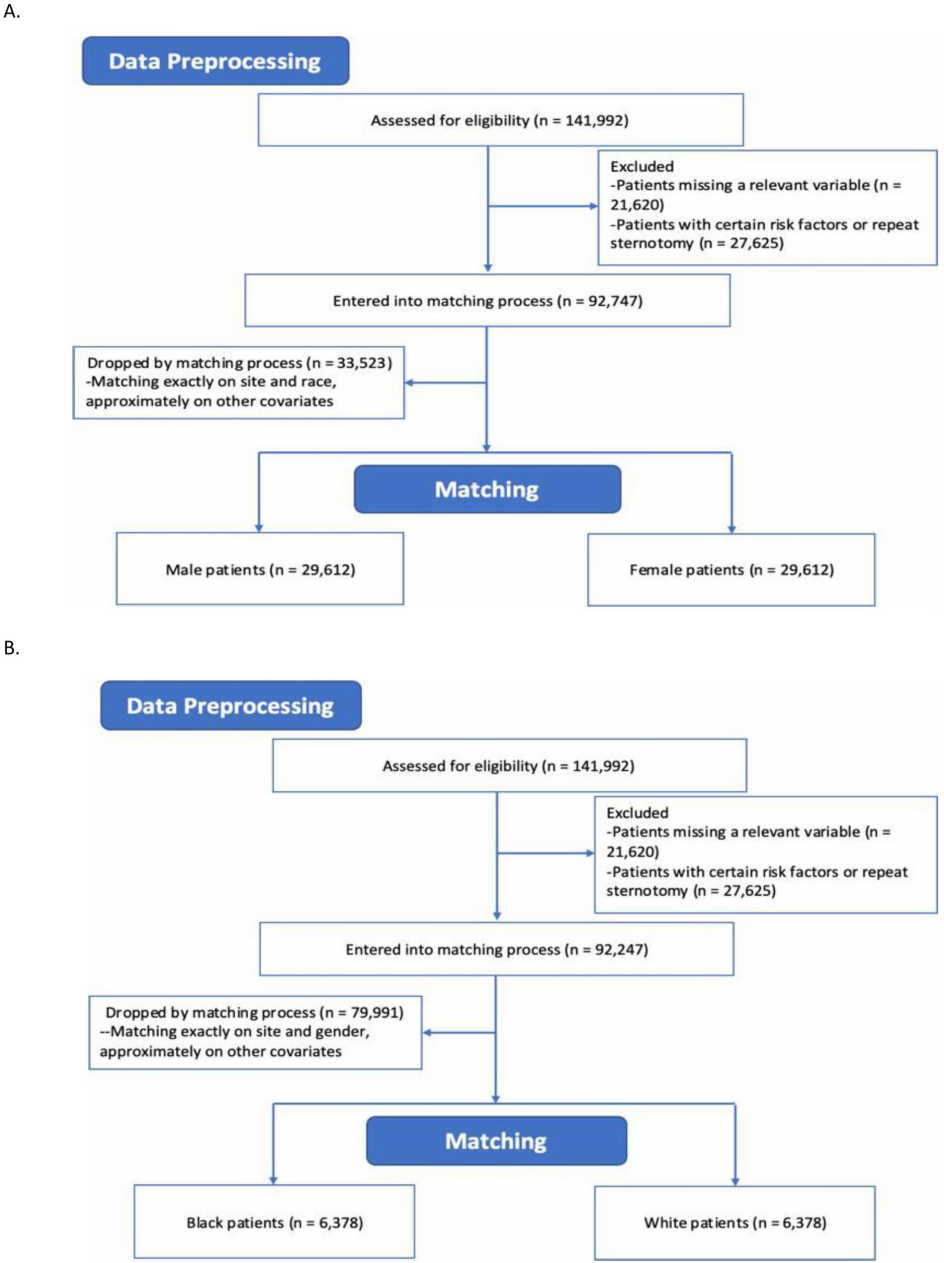
CONSORT flow diagram for the (**A**) sex and (**B**) race analysis, which shows the number of patients excluded from the analysis at each stage

Before conducting the statistical analyses, we performed several data preprocessing steps that are impactful on the inclusion/exclusion of patients in the analyses. We removed patients with missing data in any of the variables used in the analysis, including race, sex, and risk of mortality. For the racial analysis, we included only patients who identified as either White (Caucasian) or Black (African American); this excludes the potentially distinct patients with multiracial identities. We also explored a third analysis, examining socioeconomic status (SES). The details of this analysis can be viewed in the supplement. The number of patients removed by these criteria are reported flow diagrams in Fig. 1.

### Study design: matched pairs

For the two analyses, we created pair-matches between the relevant patient identity types: (i) male-vs-female sex and (ii) White-vs-Black race. The third pair-match, a comparison between high-vs-low SES, is described in the supplemental materials. In our primary analysis, patients were exactly matched within-site to ensure that between-hospital variation was not a driving factor of intra-pair outcome differences. We also performed a secondary analysis that matched by geographic region (within-region), instead of matching only within-site (e.g., some patients within a matched-pair received treatment within the same facility, but most did not). The primary analysis (within-site) considers how different types of patients are selected differentially within the same facility, whereas the secondary analysis (within-region) considers more structural forms of selection (e.g., access and barriers to receiving treatment at surgery sites).

Patients in the sex analysis were exactly matched on race, and those in the racial analysis were exactly matched on age. For all other matching covariates, which were age, body mass index, and risk of mortality, we used optimal matching with Mahalanobis distance. Tables 1 and 2 summarizes the covariates before and after matching, for the two within-site matches (Table 1) and for the two within-region matches (Table 2).

**Table 1:**
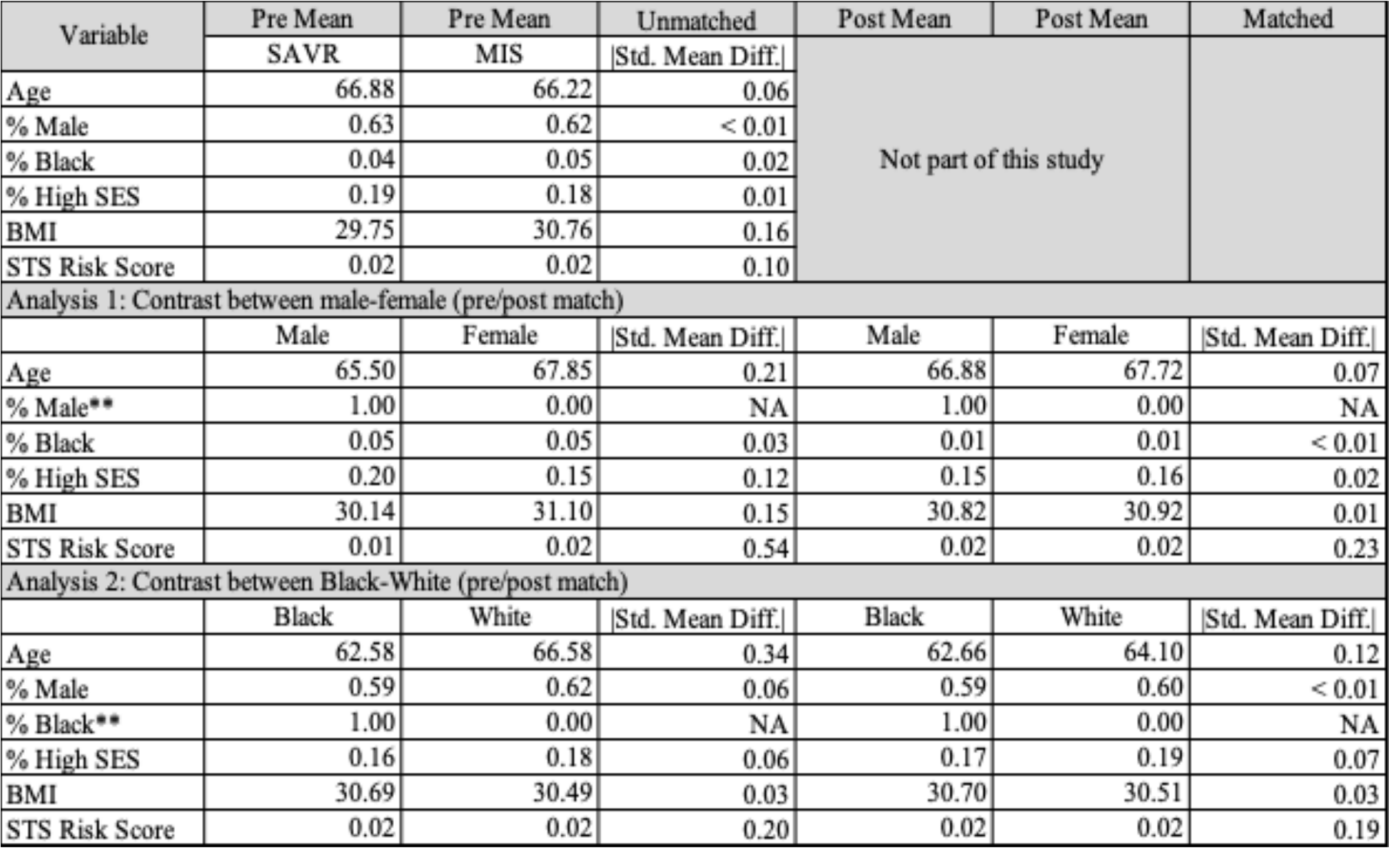
Assessment of match quality for within-site analysis. Note that covariate balance improved from pre- to post-match, except for the covariate used to create discordant identity matches (and thus intentionally separated): sex in the first match and race in the second. All other differences in either match are under the target of 0.20 SMD

**Table 2:**
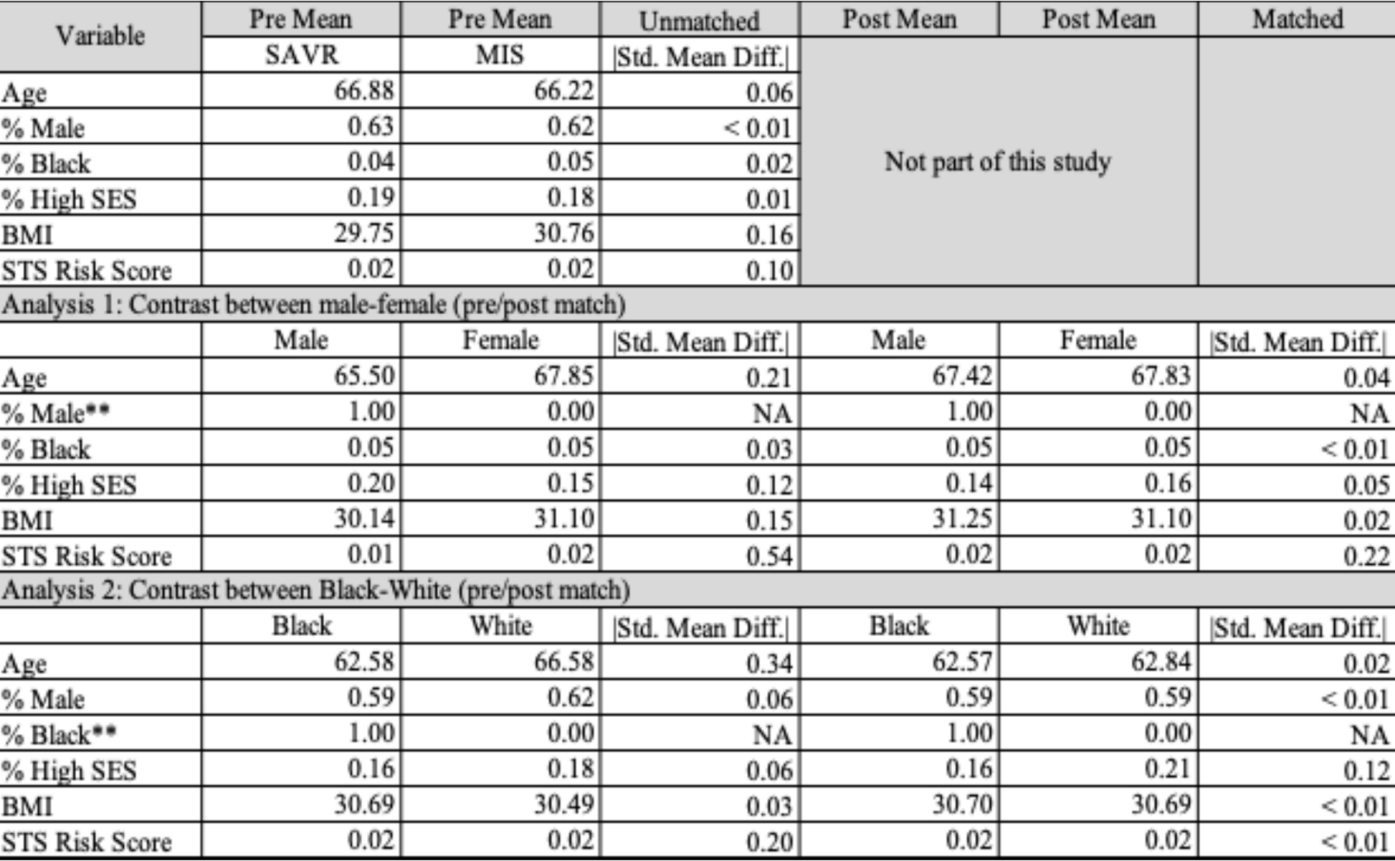
Assessment of match quality for within-region and year analysis. Note that covariate balance improved from pre- to post-match, except for the covariate used to create discordant identity matches (and thus intentionally separated): sex in the first match and race in the second. All other differences in either match are under the target of 0.20 SMD

While some matching-based study designs attempt to approximate causal effects, this study’s use of matching-based study design is non-causal and is used for “non-parametric preprocessing” [23]. The matching step is used to ensure the comparison groups have reasonable overlap in the covariate distributions (“apples to apples” clinically), to reduce the chances that extrapolation or highly leveraged values influenced the estimation of the observed differences between groups.

### Statistical analysis: generalized-linear mixed models

To understand how care was selected, we performed two related analyses. In both analyses, we pair-matched patients who had different identities and clinically relevant covariates. Tables 1 and 2 summarizes the covariates before and after matching, for the two within-site matches (Table 1) and for the two within-region matches (Table 2).

For each of the analyses, we created pair-matches between the relevant patient identity types: (i) male-vs-female sex and (ii) White-vs-Black race. We summarized these pair-matches via a generalized linear mixed model (GLMM) with a logit-link, regressing AVR surgery type on the relevant fixed effect (sex or race). Additionally, the model included random effects for STS facility and matched set identifiers. In the primary analysis, we ensured that both patients within a matched pair received their care at the same surgical facility or within-site. This type of analysis attempts to isolate variation in care to more “local” decision-making (e.g., patients in the same facility yet received different types of care). In the secondary analysis, each matched pair received care in the same geographic region. For the within- region we relaxed our matching process and allowed the pairs to have received care within the same geographic region This analysis centers on access and structural barriers to care (e.g., proximity to care).

Distinctive from several other studies, the outcome of interest in this study is the surgery received. This model is used to isolate the variation in surgery selection that can be associated with patient-identity, and the matching ensures the model is always comparing two prognostically equivalent patients with the different identity-types.

### Sub-study: summarizing differences in STS mortality risk score by race and region

**Fig. 2:**
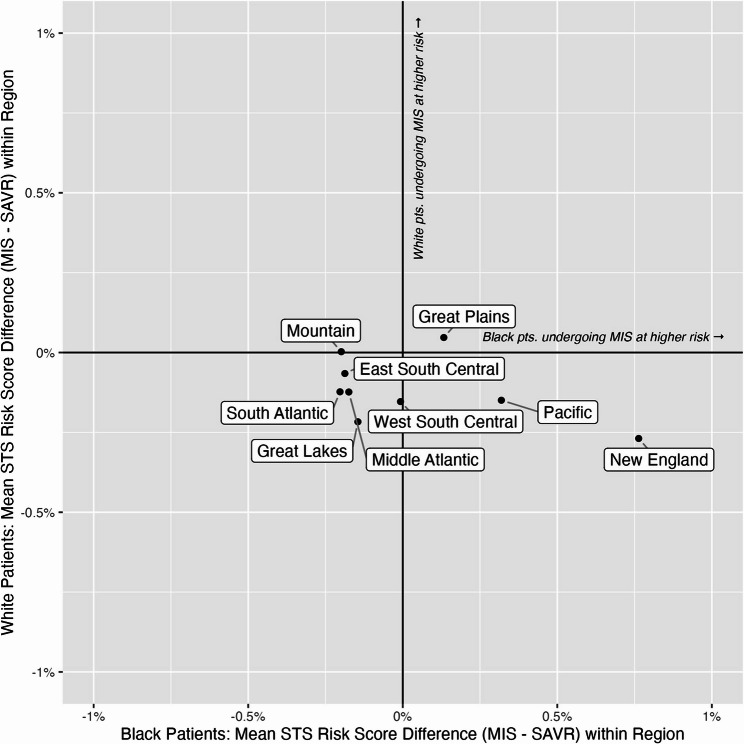
Regional variation in STS mortality risk score (STS risk score) for Black and White patients. Each point gives the difference between the average STS risk score for patients undergoing MISAVR and the average STS risk score of those undergoing SAVR, for Black (x- axis) and White patients (y-axis). For example, for the Pacific region, Black patients undergoing MISAVR had an average. For example, for the Pacific region, Black patients undergoing MISAVR had an average STS risk score of 2.26%, whereas Black patients undergoing SAVR had an average STS risk score of 1.94%, yielding a difference of 0.32%. In the same region, the average STS risk score for White patients were 1.70% and 1.57% for MISAVR and SAVR respectively, giving a difference of -0.13%

To understand geographic variation in how MISAVR and SAVR are selected, we analyzed the difference in STS mortality risk score by race. That is, without adjusting for any covariates, we summarized by region the difference in average estimated STS mortality risk score between White patients selected to MISAVR and White patients selected to SAVR. An identical summarization was also completed for Black patients selected to MISAVR vs. SAVR. This simple summary is useful for understanding the mortality risk profile of which patients are being selected to MISAVR vs. SAVR. These data are visually represented using a scatterplot by region of the United States in Fig. 2.

## Results

### Primary analyses: within same facility (within-site)

Both primary analyses identified variation in MISAVR selection between the compared groups. The prognostically equivalent pairs of patients treated within the same facility analyzed is as follows: male vs. female comparison, 29,612; and Black vs. White comparison, 6,378. The modelled estimates yielded the following patients’ odds ratio for receiving a MISAVR: female patients 1.13 times higher (p-value < = 0.005 and 95% CI between 1.08 and 1.18); and White patients 1.56 times higher (p-value < = 0.005 and 95% CI between 1.39 and 1.75).

### Secondary analysis: within-region

The secondary analysis, in which patients were matched exactly on region and year instead of surgery facility, yielded estimates similar to the within-facility analysis. The prognostically equivalent pairs of patients treated within the same facility analyzed is as follows: male vs. female comparison, 33,733; and Black vs. White comparison, 4,401. The modelled estimates yielded the following patients’ odds ratio for receiving a MISAVR: female patients 1.17 times higher (p-value < = 0.005 and 95% CI between 1.12 and 1.23; and White patients 1.22 times higher (p-value < = 0.005 and 95% CI between 1.06 and 1.40).

### Sub-study: summarizing differences in STS mortality risk score by race and region

Apart from two regions (Mountain and Great Plains), Fig. 2 demonstrates that White patients who received SAVR had a higher average STS mortality risk estimate than the White patients receiving MISAVR (e.g., data points are below the x-axis). In the Mountain region, the average STS mortality risk estimate was equivalent for MISAVR and SAVR in White patients. Also shown in Fig. 2, Black patients in the Pacific and New England regions undergoing MISAVR had higher average STS mortality risk scores than Black patients receiving SAVR. An additional interesting finding demonstrates both Black and White patients who received MISAVR in the Great Plains had higher average risk scores vs. their SAVR counterparts.

## Discussion

In our data, a pattern emerged where female and/or White patients were more likely to receive a MISAVR. Of note, though included in our supplement for reasons described in the “Limitations” section, we also found patients of higher SES were more likely to receive a MISAVR. These findings suggest non-clinical factors may influence physician decision making with respect to surgical approach. While patient advocation for a minimally invasive approach, for various reasons (i.e. cosmesis, recovery time), could explain these findings, this would only represent a small subset of patients. Moreover, there is a large cohort of patients that do not have exposure/access to the level of health literacy required for self advocation, highlighting the need to examine health equity. Health equity, as defined by the Centers for Disease Control and Prevention (CDC), is “the state in which everyone has a fair and just opportunity to attain their highest level of health” ([24] (e1)). Lack of health equity is a leading contributor to health disparities, which costs over $93 billion a year in medical care [25]. According to the CDC, certain racial/ethnic minority groups, persons of lower SES, and/or persons living in remote areas have a higher likelihood of encountering multiple barriers of access to health care, leading to poor patient health and health outcomes [24]. A literature review examining disparities in healthcare access across multiple surgical subspecialties conducted by de Jager et al. found a lack of equitable access to quality care [26]. Using an example of surgical treatment for breast cancer, de Jager et al. describes how racial/ethnic minorities were more likely to have [26]: 1. care by lower quality providers due to accessibility, 2. a delay in determination of need for- and execution of- surgical treatment, and 3. inadequate surgical treatment and follow-up care. de Jager et al. reported similar findings for subspecialties such as vascular and thoracic surgery [26].

Other key factors that can influence “local” decision making includes financial barriers (e.g., reimbursement) and surgeon experience with minimally invasive surgery (MIS) techniques [27, 28]. Surgeon experience is largely based on exposure during their training. In Tolis’s examination of current cardiac surgical training, he surmised that cardiac residents may not have sufficient experience to enter the workforce, hypothesizing initiation of mandatory work hour restriction (e.g., varying surgical case exposure) and/or increase in case complexity could be contributing factors [29] Using MIS techniques, can increase the complexity of a case, as these approaches are not practiced ubiquitously in cardiac surgery resulting in a trainee’s exposure to MIS techniques being unpredictable. Thus, if a resident trains at a low/no volume institution, they will have little to no experience with MIS techniques. As stated by Wennberg et al., when examining variations in medical care, “The attitude of the physicians at that hospital therefore has a strong influence on the rate of a given procedure in the surrounding area” ([30] (pg120)). Meaning, physicians will perform/teach procedures that they are most familiar with, which can result in an uneven pipeline of specially trained surgeons; propagating institutional variation due to:

1. Shortage of MIS Surgeons per facility –surgical approach dependent on patient referral to a specific surgeon within the facility comfortable with MIS approaches. Surgeons who commonly utilize MIS techniques are more likely to consider this approach vs. a surgeon with less experience within the same institutions.
2. Disproportionate technology diffusion – without appropriately trained surgeons on staff helming innovation, health organizations will not have the resources to offer new techniques [28].

Our secondary analysis, within- region, yielded similar results to our primary analysis. This analysis elaborates on how patients selected into different facilities, within the same region, impacts care received. Regional practice variation is a longstanding problem worldwide [27] Birkmeyer et al. reviewed Medicare data from the Dartmouth Atlas of Healthcare and reported several surgical procedures, including coronary artery bypass surgery, exhibited a four- to five -fold variation across regions [28]. When variation is significant, it can indicate inappropriate utilization of medical services and lead to excessive medical costs and poor patient outcomes [27] Additionally, with differential procedural reimbursements by region, physicians trained in MIS techniques will likely gravitate to specific regions, further propagating regional shortages of experienced MIS surgeons [28].

As previously mentioned, a third analysis was completed exploring differences between patients of low and high SES. We did not have access to patient-specific SES information; therefore we used an imprecise proxy measure which was insurance type (private insurance vs. Medicare). A difference was found between groups (see supplement). Though we believe an analysis of this type – i.e., exploring variation associated with SES – would make important contributions to the literature, the lack of patient-specific information on their SES limits the accuracy of our exploratory analysis (see *Limitations* section). We hope future research endeavors using more precise tools, such as the CDC’s Social Vulnerability Index, will be undertaken as these findings could have important implications with respect to health disparities and equity [31].

Though we illustrated a demographically linked selection pattern exists in our primary and secondary analysis, what remains unclear is why the observed selection pattern exists. While the literature has not demonstrated differences in major outcomes like survival, MISAVR may have particular benefits for patients who are more resource constrained. For example, if a patient is able to have a faster post-surgical recovery, it may portend a substantial socioeconomic benefit in at-risk patient populations (e.g., less time away from work - leading to increased jobs security, financial stability, etc.), highlighting the need for further research and importance to determine causation.

### Limitations

A more thorough comparison between the two types of study designs would address any differential covariate distributions between the analysis populations (e.g., issues of generalizability and extrapolation). Future prospective analyses could mitigate some limitations of the current study. For instance, we used data from the STS database. Variables not collected by the STS and observations from institutions that do not submit data to the STS database was not included. We also excluded the comparison of patients who underwent a TAVR procedure, as this data is not available in the STS database. While we matched patients on BMI, age, and STS mortality risk score, patient factors that were not used or included in the calculation of the STS risk score (e.g., body habitus, aortic calcification, chest wall deformity, etc.) were not accounted for during our patient matching process and could influence surgical decision making [32]. Additionally, we performed a third analysis (only reported in the supplement) which approximated SES using medical insurance. Utilizing patient-specific information such as zip code of patient residence, level of education, employment status, or income would improve accuracy when estimating patient SES. We hope future analyses can perform a rigorous analysis of how SES relates to surgical selection. Finally, surgeon information (e.g., surgical training, location, case volume, etc.) was unavailable and may provide insight for interpreting data with respect to case variation both within-site and -region.

## Conclusions

After accounting for medically relevant patient factors, our study isolated a pattern demonstrating variation in selection to MISAVR vs. SAVR, tracking patient sex and race. These findings suggest patient candidacy for surgical approach, even within a facility, is guided by non-medical patient characteristics. While making definitive attributions will be challenging, we hope this manuscript serves as a foundation for future research efforts focused on care allocation and wider accessibility innovative surgical techniques, providing empirical evidence for developing targeted interventions for reducing healthcare inequities.

## Supplementary Information

Supplementary Material 1.

## Data Availability

The data in this manuscript was provided by a third party (The Society of Thoracic Surgeons’ National Database Participant User File Research Program) under legal contract and by permission. Data will be shared upon reasonable request to the corresponding author with permission of the third party listed.

## Glossary

AS: Aortic stenosis
AVR: Aortic valve replacement
BMI: Body mass index
CDC: Centers for Disease Control and Prevention
GLMM: Generalized linear mixed model
h/o: history of
MIS: Minimally invasive surgery
MISAVR: Minimally invasive surgical aortic valve replacement
PUF: Participant User File Research Program
SAVR: Traditional full sternotomy aortic valve replacement
SES: Socioeconomic status
STS: The Society of Thoracic Surgeons
TAVR: Transcatheter Aortic Valve Replacement
URM: Under-represented minority
