# Supplementary material for: Trends in Open Aortic Valve Surgery in the United States: Descriptive Analysis of Sociodemographic Factors in Patient Selection
Source: BMC Surg. 2026 Feb 25;26:235. doi: 10.1186/s12893-026-03621-9 (PMC13041147; doi:10.1186/s12893-026-03621-9)
Supplement: Supplementary file 1 — Supplementary Material 1. [file 12893_2026_3621_MOESM1_ESM.docx]

Supplemental Material

**Methods**

As an exploratory analysis, we conducted a third analysis looking at socioeconomic (SES) factors. Unfortunately, we only had access to a poor quality proxy for SES: insurance type. Previous research has used insurance category as a proxy measure for patient socioeconomic status (SES) (e.g., Medicare and private insurance vs Medicaid and uninsured cohorts).^6,7,21^ Patient insurance category may contribute to both a provider’s decision to refer a patient for cardiac surgery and a patient’s decision to accept the referral for reasons of cost, availability of procedure options, and availability of post- procedure care.^6^ We report this third analysis in the supplement because we believe insurance-type as a proxy for SES is not strong. We would have preferred to use the CDC’s Social Vulnerability Index (SVI) as the proxy for SES (https://www.atsdr.cdc.gov/place-health/php/svi/index.html). Unfortunately for this analysis, the SVI requires linking patients using their Zip Code information, which is not available in the STS’s PUF version. We include this SES analysis in the supplement in hopes that it may support future research in this direction.

*Inclusion/Exclusion*

We used all of the same inclusion/exclusion criteria for this SES analysis as was used in the sex and race analysis. See Figure S1 for the results of the inclusion/exclusions steps for the SES analysis.

*Preprocessing Method*

We removed patients with missing data in any of the variables used in the analysis, including race, gender, and risk of mortality. For the SES analysis, we coded people as low SES if they had no health care, Medicaid, or only charitable care, and high SES if they had commercial health insurance or were part of a health maintenance organization. For the SES analysis, patients with any other health insurance types were removed (e.g., Medicare).

*Study design: matched pairs*

We used the same design strategy for the SES analysis as we did for sex and race. For the SES analysis, we included race and gender as additional covariates in the approximate matching. *Tables S1 and S2* summarizes the covariates before and after matching, for within-site match (*Table S1*) and for the within-region match (*Table S2*).

While some matching-based study designs attempt to approximate causal effects, this study’s use of matching-based study design is non-causal and is used for “non-parametric preprocessing”.^23^ The matching step is used to ensure the comparison groups have reasonable overlap in the covariate distributions (“apples to apples” clinically), to reduce the chances that extrapolation or highly leveraged values influenced the estimation of the observed differences between groups.

**Results**

We report all three analyses here in the supplement in order to facilitate comparison.

*Primary analyses: within same facility (within-site)*

All three primary analyses identified variation in MISAVR selection between the compared groups. The prognostically equivalent pairs of patients treated within the same facility analyzed is as follows: male vs female comparison, 29,612; Black vs White comparison, 6,378; and high vs low SES patient comparison, 1,844. The modelled estimates yielded the following patients’ odds ratio for receiving a MISAVR: female patients 1.13 times higher (p-value <= 0.005 and 95% CI between 1.08 and 1.18); White patients 1.56 times higher (p-value <= 0.005 and 95% CI between 1.39 and 1.75); and high SES patients 1.31 times higher (p-value <= 0.005 and 95% CI between 1.10 and 1.56).

*Secondary analysis: within-region*

The secondary analysis, in which patients were matched exactly on region and year instead of surgery facility, yielded estimates similar to the within-facility analysis. The prognostically equivalent pairs of patients treated within the same facility analyzed is as follows: male vs female comparison, 33,733; Black vs White comparison, 4,401; and high vs low SES patient comparison, 3,266. The modelled estimates yielded the following patients’ odds ratio for receiving a MISAVR: female patients 1.17 times higher (p-value <= 0.005 and 95% CI between 1.12 and 1.23; White patients 1.22 times higher (p-value <= 0.005 and 95% CI between 1.06 and 1.40); and high SES patients 1.28 times higher (p-value <= 0.005 and 95% CI between 1.09 and 1.51).


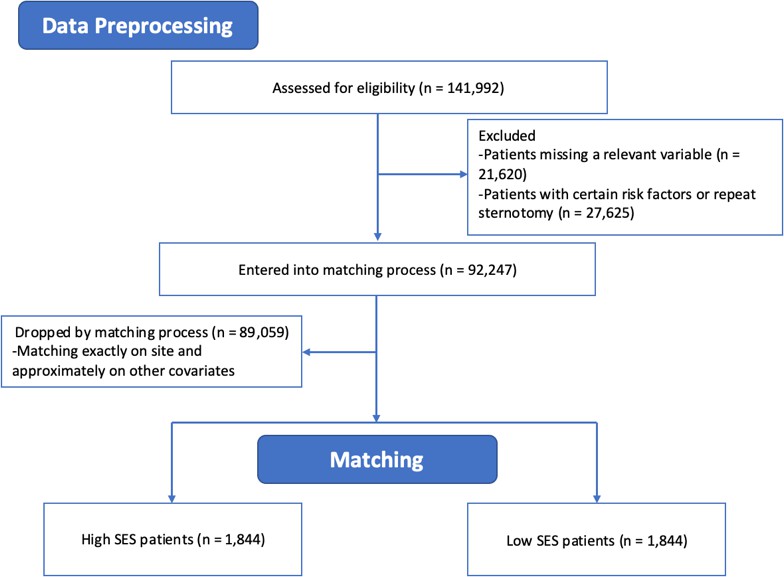


Figure S1. **CONSORT flow diagrams** for socioeconomic status analysis, which show the number of patients excluded from the analysis at each stage.

Table S1. **Assessment of match quality for within-site analysis**. Note that covariate balance improved from pre- to post-match, except for the covariate used to create discordant identity matches (and thus intentionally separated): SES in this match. All other differences in either match are under the target of 0.20 SMD.

Table S2. **Assessment of match quality for within-region and year analysis**. Note that covariate balance improved from pre- to post-match, except for the covariate used to create discordant identity matches (and thus intentionally separated): SES in this match. All other differences in either match are under the target of 0.20 SMD.
